# Outcomes and prognoses of patients with ovarian cancer using bevacizumab: 6-year experience in a tertiary care hospital of northern Taiwan

**DOI:** 10.1371/journal.pone.0175703

**Published:** 2017-05-03

**Authors:** Wei-Chun Chen, Jiantai Timothy Qiu, Chyong-Huey Lai, Huei-Jean Huang, Cheng-Tao Lin, Min-Yu Chen, Hung-Hsueh Chou, Kuan-Gen Huang, Ting-Chang Chang

**Affiliations:** 1 Department of Obstetrics and Gynecology, Chang Gung Memorial Hospital. Taoyuan, Taiwan; 2 Chang Gung University College of Medicine, Taoyuan, Taiwan; 3 Gynecologic Cancer Research Center, Chang Gung Memorial Hospital. Taoyuan, Taiwan; 4 Department of Biomedical Sciences, Chang Gung University, Taoyuan, Taiwan; Zhejiang University School of Medicine, CHINA

## Abstract

**Purpose:**

Bevacizumab (BEV) has been used for ovarian cancer (OC) for years in Taiwan, but the associated data related to outcome is scant. This retrospective study reviewed patients with OC treated with BEV and analyzed their results.

**Patients and methods:**

All patients with OC treated with BEV from 2009 to 2015 in the Linkou branch of Chang Gung Memorial Hospital in Northern Taiwan were included. According to the means of administration, the patients were classified into 6 groups as follows: A—BEV plus chemotherapy (C/T) for initial platinum-resistant (PR) recurrent OC, B—BEV plus C/T for initial platinum-sensitive (PS) recurrent OC, C—BEV alone for recurrent OC, D—BEV plus 1^st^ adjuvant C/T, E—BEV plus neoadjuvant C/T, and F—intraperitoneal (IP) BEV. Progression-free survival (PFS), overall survival (OS), hazard ratios (HRs), overall response rate (ORR), and mean number of BEV cycles were analyzed for groups A to E. Clinical improvement of ascites was assessed for group F.

**Results:**

A comparison of early use (only one round of prior C/T) versus late use (multiple rounds of prior C/T) in patients of groups A and B showed a superior PFS (8.27 vs. 3.67, p = 0.037) in the early use group. No significant differences were found between groups A and B (PFS: 4.24 vs. 4.17 months, p = 0.690; OS: 10.06 vs. 9.93 months, p = 0.819; mean BEV cycles: 4.63 vs. 5.0 p = 0.992; ORR: 48.1% vs. 53.5%, p = 0.425). Comparing the response and non-response subgroups of patients in groups A and B, a better outcome was associated with endometrioid type cell (HR = 0.28, p = 0.008), good ECOG performance status (HR = 0.51, p = 0.005), and lack of ascites (HR = 0.67, p = 0.004). Comparing group C with groups A plus B, the BEV alone group had a poorer PFS (1.02 VS. 4.19, p = 0.04) and OS (1.42 VS. 9.99 p = 0.001) than the BEV plus C/T group. In group F, a good clinical benefit rate (85.6%) of ascites improvement was noted. Two patients had grade 5 gastrointestinal bleeding and venous/arterial thromboembolic events after administration of BEV. Grade 3 neutropenia and thrombocytopenia occurred more frequently in our study.

**Conclusion:**

Early use of BEV combined with chemotherapy had a significant benefit in PFS for patients with recurrent OC. BEV plus chemotherapy was better than BEV alone for recurrent OC. In addition, IP BEV was helpful for improving clinical ascites.

## Introduction

Ovarian cancer is one of the most common gynecologic cancers, with nearly 22,000 new-onset cases and 14,000 deaths in the United States in 2015.[1] In Taiwan, there are nearly 1,000 new cases and 400 deaths per year.[2] Despite treatment with multiple, newly developed therapeutic agents, the prognosis of ovarian cancer remains poor, and the 5-year survival rate of ovarian cancer is approximately 46%.[1] Angiogenesis promotes tumor growth and metastasis, and anti-vascular endothelial vascular factor (VEGF) has a potential tumor suppression effect.[3–7] Bevacizumab is a humanized monoclonal antibody directed against VEGF-A as target therapy.[8] After its initial approval by the Food and Drug Administration (FDA) in 2004 for unresectable colorectal cancer, its indication for the treatment of different cancers has been accepted.[9, 10] There are several current published reports from major clinical trials of bevacizumab use in ovarian cancer. GOG-0218 and ICON7 reported bevacizumab use in combination with front-line adjuvant chemotherapy and as maintenance.[11–13] For platinum-sensitive recurrent ovarian cancer, OCEANS and GOG-0213 studied bevacizumab combined with platinum-based chemotherapy and as maintenance.[14–16] AURELIA studied bevacizumab in combination with non-platinum chemotherapy and as maintenance in platinum-resistant recurrent ovarian cancer.[17] All of the above major clinical trials have illustrated prolonged progression-free survival compared with placebo or chemotherapy alone. However, no overall survival advantage was found in the bevacizumab arm except GOG-0213 study showing 5 months of benefit in Bevacizumab containing group comparing to chemotherapy alone. Because only AURELIA reached the endpoint of progression-free survival, since 2014, the FDA has only approved bevacizumab for platinum-resistant recurrent ovarian cancer in gynecologic oncology.[9, 10] On Dec 6th, 2016, FDA also approved its indication of platinum-sensitive recurrent ovarian cancer based on the results of GOG-0213.[18]

In Taiwan, bevacizumab has been used for ovarian cancer for years, but the associated data have not yet been published. Therefore, we present our experience of bevacizumab for ovarian cancer in the Linkou branch of Chang Gung Memorial Hospital in northern Taiwan.

## Methods

### Patients and study design

This is a retrospective study analyzing data from the Linkou branch of Chang Gung Memorial Hospital from 2009 until 2015. The study was approved by the local ethics committee (IRB 105-4036C). All patients with ovarian cancer treated with bevacizumab were enrolled. The clinical and pathological data were obtained from medical records. The patient records/information were anonymized and de-identified prior to analysis. All the enrolled patients were discussed and reviewed at initial diagnosis or while newly recurrence in our tumor board conference held weekly. Besides, as the international guideline and our team consensus suggested, all the patients received close follow-up every 1–2 months for the initial 6 months, 2–3 months from 6 months to 2 years after treatment, every 3–6 months until 5 years, and then annually thereafter.[19] Tumor markers were checked every visit, and computed tomography (CT) or magnetic resonance imaging (MRI) were arranged every year for 2 years after treatment, while any suspected tumor markers elevation, or any clinically needed.

**Fig 1:**
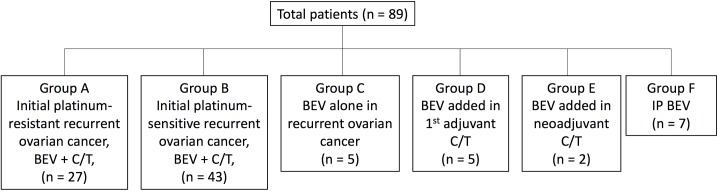
Patient classification. BEV, bevacizumab; C/T, chemotherapy; IP, intraperitoneal.

All patients were classified into 6 groups (Fig 1): initial platinum-resistant recurrence with chemotherapy-combination group (group A), initial platinum-sensitive recurrence with chemotherapy-combination group (group B), recurrence with bevacizumab alone group (group C), bevacizumab combined with first adjuvant chemotherapy group (group D), bevacizumab combined with neoadjuvant chemotherapy group (group E), and intraperitoneal bevacizumab group (group F).

The primary outcomes of interest were progression-free survival and overall survival. Other outcomes included toxicity, safety, overall response to therapy, number of bevacizumab cycles, clinical improvement of ascites. There were no routine imaging follow-up studies including computed tomography (CT) or magnetic resonance imaging (MRI) before or after bevacizumab treatment in any case. Response and progress were defined based on cancer antigen 125 (CA-125) criteria from the Gynecologic Cancer InterGoup (GCIG): a response was defined as when CA-125 decreased to half of its initial level and persisted for at least 28 days; and progress was defined as when CA-125 was more than twice the upper limit of reference if the nadir was within normal limits, or when it was more than twice the nadir level if the nadir was greater than the upper limit of the reference.[20] The clinical benefit rate of ascites improvement was evaluated according to the daily progress notes and nursing records because there was no routine abdominal circumference assessment for all women.

Safety and adverse effects were monitored and recorded in each case until 30 days after the last administration of bevacizumab. The extent of severity was evaluated and assessed by the Common Terminology Criteria for Adverse Events (CTCAE).[21] All grade 5 adverse effects are also included in our survey and article.

### Statistical analysis

The progression-free survival and overall survival were analyzed by Kaplan-Meier curves. The differences in progression-free survival and overall survival among groups were evaluated with log-rank tests. The hazard ratios of different influencing factors of the response and non-response groups were calculated using Cox regression hazards models. Data between two different groups were compared using the paired t-tests and Chi-square tests.

## Results

### Patients

**Fig 2:**
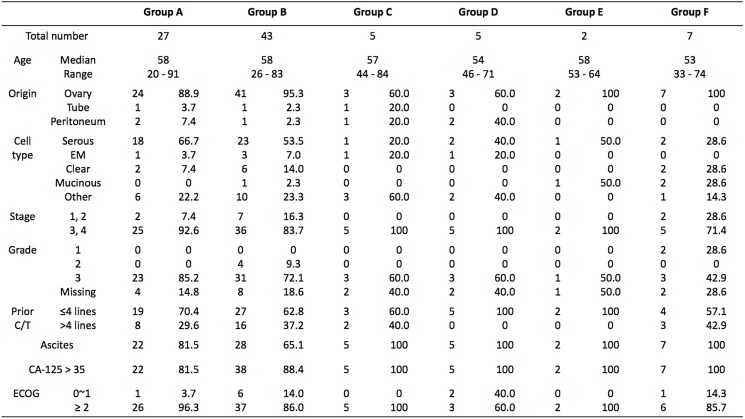
Baseline patient characteristics. C/T, chemotherapy; EM, endometrioid.

The data of 89 women treated at the Linkou branch of Chang Gung Memorial in northern Taiwan between 2009 and 2015 were collected. As described in the previous section, all of the enrolled patients were classified into 6 groups. There were 27 women in group A, 43 in group B, 5 in group C, 5 in group D, 2 in group E, and 7 in group F. The patients in both groups A and B had recurrent ovarian cancer and were treated with a combination of bevacizumab and chemotherapy. Their characteristics are as follows: the median age was 58 years, 93% had ovarian-origin cancer, 87% had a late FIGO (The International Federation of Gynecology and Obstetrics) stage (stage 3 or 4), 77% had grade 3 tumor cells, 71% had obvious ascites, 86% had CA-125 over the upper limit of reference, and 90% had ECOG (Eastern Cooperative Oncology Group) performance status greater than 1. By histologic tumor type, 59% of the patients have serous type, 6% have endometrioid type, and 11% have clear-cell type. Regarding prior regimens of chemotherapy, 66% of the women had had no more than 4 rounds of chemotherapy regimens before bevacizumab administration, and only 11% had had only one regimen before bevacizumab. The patients’ basic characteristics are shown in Fig 2.

### Study treatments received

**Fig 3:**
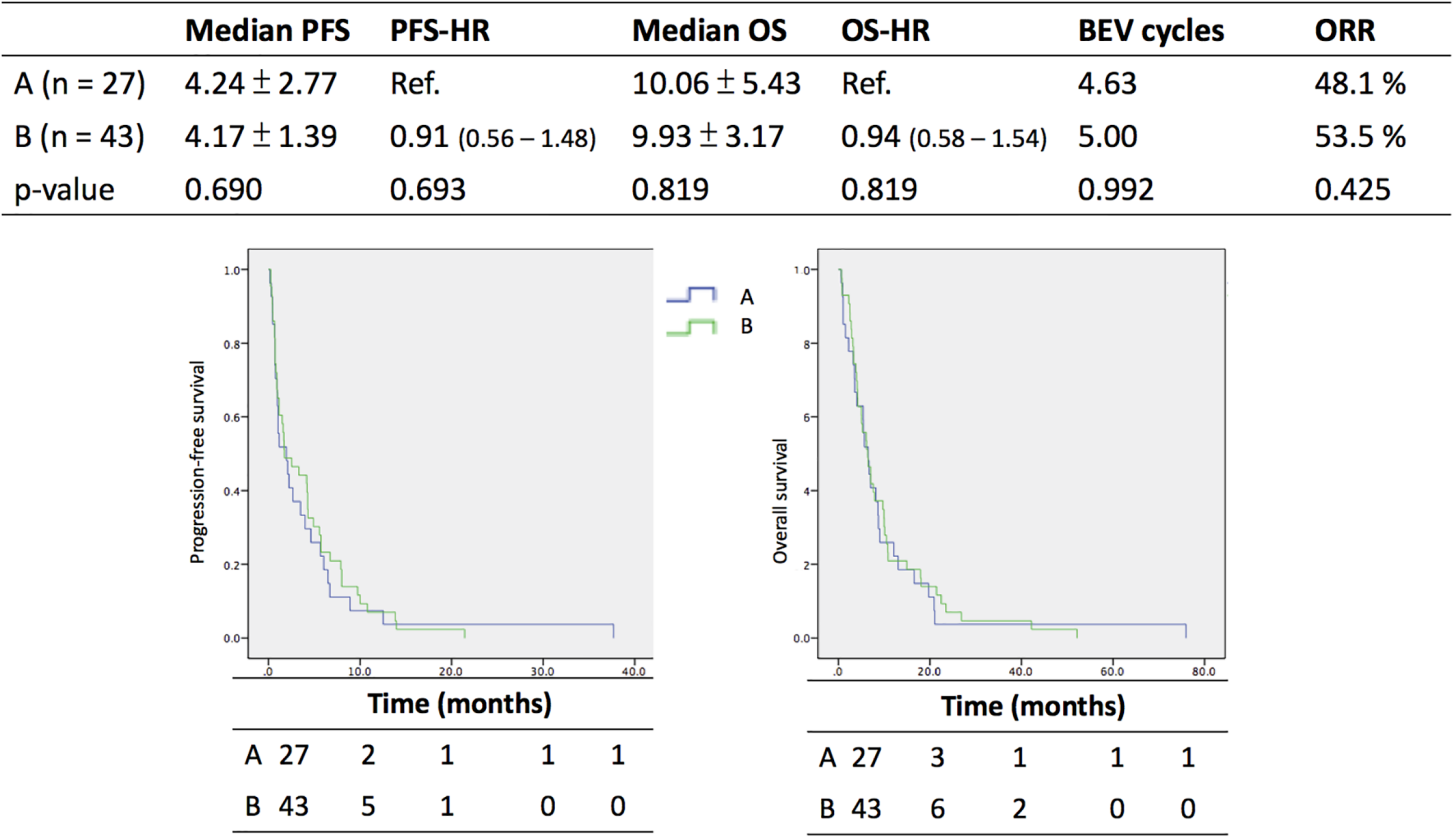
Median PFS and OS of group A and B. PFS, progression-free survival; OS, overall survival; HR, hazard ratio; ORR, overall response rate; Ref., reference; BEV, bevacizumab.

A comparison of groups A and B, which include all of the enrolled women who received chemotherapy in combination with bevacizumab, is shown in Fig 3. In groups A and B, the median progression-free survival was 4.24 ± 2.77 and 4.17 ± 1.39 months, respectively; the median overall survival was 10.06 ± 5.43 and 9.93 ± 3.17 months, respectively; the overall response rate was 48.1% and 53.5%, respectively; and the mean number of courses of bevacizumab administration was 4.63 and 5.0 cycles, respectively. None of the above clinical endpoints had a significant p-value.

Because there were no obvious differences between groups A and B, the data of both were combined into one category that included all of the patients with recurrent ovarian cancer who were treated with a combination of chemotherapy and bevacizumab. Then, a comparison of the response group and the non-response group was performed. As shown in Tables 1 and 2, the following results were obtained: the progression-free survival between the response group and non-response group was 7.256 and 0.952 months, respectively; and the overall survival was 14.315 and 5.418 months, respectively. Both progression-free survival and overall survival had significant p-values. In the response group and the non-response group, the median age was 56 and 60 years old, respectively; the mean number of courses of bevacizumab administration was 6.17 and 3.47 cycles, respectively; and the mean number of prior regimens of chemotherapy was 3.22 and 4.68 rounds, respectively. Additionally, in the response group and the non-response group, the median initial CA-125 level at the time of bevacizumab administration was 1997.13 U/ml and 2358.15 U/ml, respectively; the percent of patients with an initial FIGO stage of 3 or 4 was 83.3% and 91.2%, respectively; and 72.2% and 82.4%, respectively, were grade 3 tumor cells. There were no significant p-values in the above comparisons. Analysis of tumor origin showed that in the response group and the non-response group, 88.9% and 97.1% of patients had tumors of ovarian origin, respectively, and the hazard ratio of ovarian origin was 3.04 (95% confidence interval: 1.27–7.27) compared with tubal origin; the p-value for this was significant. In the response and non-response groups, based on histologic tumor type, 0% and 2.9% of tumors, respectively, were mucinous type; 4% and 0%, respectively, were endometrioid type; and the hazard ratios of the mucinous type and the endometrioid type were 20.77 (95% confidence interval: 3.0–143.7) and 0.28 (95% confidence interval: 0.11–0.72), respectively. Both of these hazard ratios had significant p-values. Analysis of ECOG performance status showed that in the response group and the non-response group, 16.7% and 2.9%, respectively, of the women had a better performance status (ECOG 0 and 1), and the hazard ratio for better ECOG status was significant at 0.51 (95% confidence interval: 0.32–0.82). Regarding clinical ascites, 41.7% of the response group and 14.7% of the non-response group had ascites, and the hazard ratio was significant at 0.67 (95% confidence interval: 0.51–0.88) for patients without ascites. In addition, the dosage of bevacizumab per week and the chemotherapy regimen were evaluated; in the response group and the non-response group, 27.8% and 20.6% of patients, respectively, were administered a low dose (0–50 mg per week), and 30.6% and 38.2%, respectively, were administered an intermediate dose (76–100 mg per week). There were no significant p-values associated with either the dose of bevacizumab per week or the chemotherapy regimen.

**Table 1:** Parameters analysis of response and non-response patients in group A and B. **PFS.** progression-free survival; CI, confidence interval.

		Response group	Non-response group	PFS-Hazard ratio	p-value
		(n = 36)	(n = 34)	(95% CI)	
Stage	1, 2	6 (16.7%)	3 (8.8%)	Reference	0.249
	3, 4	30 (83.3%)	31 (91.2%)	1.57 (0.73–3.35)	
Grade	3	26 (72.2%)	28 (82.4%)	Reference	0.872
	1 & 2	1 (2.8%)	3 (8.8%)	1.06 (0.52–2.15)	0.252
	Missing	9 (25%)	3 (8.8%)	0.73 (0.43–1.25)	
Site	Tube	2 (5.6%)	1 (2.9%)	Reference	0.013
	Ovary	32 (88.9%)	33 (97.1%)	3.04 (1.27–7.27)	0.952
	Peritoneum	2 (5.6%)	0	1.03 (0.37–2.88)	
Histology	Serous	18 (50%)	23 (67.6%)	Reference	0.002
	Mucinous	0	1 (2.9%)	20.77 (3.0–143.7)	0.008
	Endometrioid	4 (11.1%)	0	0.28 (0.11–0.72)	0.06
	Clear	5 (13.9%)	3 (8.8%)	0.46 (0.21–1.03)	0.07
	Other	9 (25%)	7 (20.6%)	0.54 (0.28–1.05)	
ECOG	≥ 2	30 (83.3%)	14 (97.1%)	Reference	0.005
	0 & 1	6 (16.7%)	1 (2.9%)	0.51 (0.32–0.82)	
Ascites	Yes	21 (58.3%)	29 (85.3%)	Reference	0.004
	None	15 (41.7%)	5 (14.7%)	0.67 (0.51–0.88)	
Progression-free survival		7.256	0.952		< 0.005
Overall survival		14.315	5.418		< 0.005
Age		56	60		0.812
Avastin cycles		6.17	3.47		0.094
Recur after platinum		12.91	10.82		0.656
Prior chemo regimen		3.22	4.68		0.194
CA-125 level		1997.13	2358.15		0.706

**Table 2:** Bevacizumab dosage analysis and combined chemotherapy regimens in group A and B. PFS, progression-free survival; OS, overall survival; CI, confidence interval.

**Avastin dosage per week (mg)**	**Response group**	**Non-response group (n = 34)**	**Median PFS**	**PFS-Hazard ratio (95% CI)**	**Median OS**	**OS-Hazard ratio (95% CI)**
	(n = 36)		**(range)**		**(range)**	
0–50	10 (27.8%)	7 (20.6%)	4.44 (1.91–6.98)	Reference	8.65 (4.98–12.32)	Reference
51–75	2 (5.6%)	6 (17.6%)	6.37 (0–15.23)	0.92 (0.38–2.23)	14.96 (0–32.19)	0.72 (0.29–1.77)
76–100	11 (30.6%)	13 (38.2%)	3.50 (1.89–5.10)	1.17 (0.63–2.20)	11.5 (6.35–16.66)	0.83 (0.44–1.55)
101–150	5 (13.9%)	4 (11.8%)	3.39 (1.45–5.33)	1.34 (0.59–3.03)	9.05 (4.86–13.24)	0.97 (0.43–2.17)
151–200	5 (13.9%)	3 (8.8%)	4.38 (1.19–7.54)	1.03 (0.44–2.40)	6.45 (2.31–10.60)	1.45 (0.62–3.38)
> 200	3 (8.3%)	1 (2.9%)	4.43 (0–8.88)	0.99 (0.33–2.99)	5.90 (7.14–12.84)	1.48 (0.49–4.45)
**Chemo regimen**	**Response group (n = 36)**	**Non-response group (n = 34)**	**Median PFS**	**Median OS**		
			**(range)**	**(range)**		
5-FU	8 (22.2%)	13 (38.2%)	4.43 (0.81–8.06)	11.30 (4.48–18.12)		
Lipodox	10 (27.8%)	8 (23.5%)	3.76 (1.98–5.53)	7.98 (3.74–12.22)		
Platinum	15 (41.7%)	6 (17.6%)	5.59 (3.84–7.34)	10.91 (6.65–15.18)		
Paclitaxel	15 (41.7%)	7 (20.6%)	5.42 (3.21–7.63)	9.29 (6.27–12.30)		
Gemcitabine	3 (8.3%)	2 (5.9%)	3.21 (0.88–5.53)	5.67 (4.80–6.54)		

**Fig 4:**
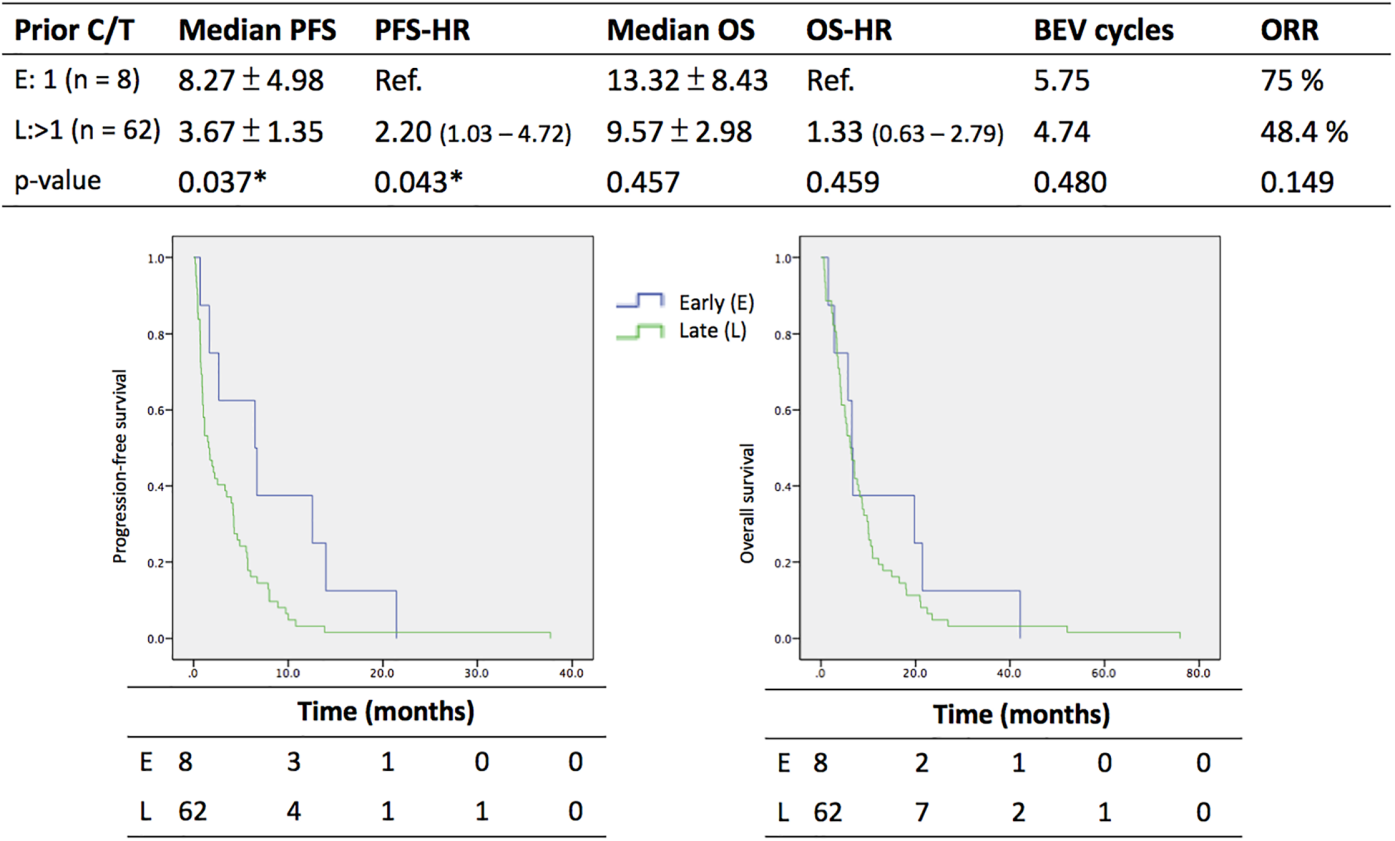
Outcome analysis of early and late use of Bevaziumab in group A and B. E, early group; L, late group; C/T, chemotherapy; PFS, progression-free survival; OS, overall survival; HR, hazard ratio; ORR, overall response rate; Ref., reference; BEV, bevacizumab.

In the comparison of early and late administration of bevacizumab in combination with chemotherapy for recurrent ovarian cancer, patients with only one prior chemotherapy regimen (early administration group) were compared with patients with multiple previous regimens (late administration group), and the results are shown in Fig 4. The median progression-free survival of the early and late groups was 8.27 ± 4.98 and 3.67 ± 1.35 months, respectively, and the hazard ratio of progression-free survival for late administration was 2.20 (95% confidence interval: 1.03–4.72); the above data had a significant p-value. In contrast, the median overall survival of the early and late groups was 13.32 ± 8.43 and 9.57 ± 2.98 months, respectively; the mean number of cycles of bevacizumab administration was 5.75 and 4.74 cycles, respectively; and the overall response rate of bevacizumab was 75% and 48.4%, respectively. No significant p-values were found in the survey of these last three endpoints.

In group C, those with recurrent ovarian cancer treated with bevacizumab alone, the poor progression-free survival (1.02 ± 0.64 months) and overall survival (1.42 ± 0.97 months) are shown in Table 3. The overall response rate was only 40%, and only 1.60 mean cycles of bevacizumab administration were administered. As shown in Table 3, in groups D and E, there was a 100% response rate with a much higher mean number of cycles of bevacizumab administration (15.20 and 7.50, respectively). In addition, good progression-free survival (16.36 ± 2.83 months in group D; 4.03 ± 5.94 months in group E) and overall survival (32.27 ± 14.48 months in group D; 9.650 ± 5.06 months in group E) were also found, and these data might be even better because many patients (60% in group D; 100% in group E) are still alive.

**Table 3:** Outcome analysis of group C, D, and E. PFS, progression-free survival; OS, overall survival; ORR, overall response rate; BEV, bevacizumab.

	Number	Median PFS	Median OS	ORR	Mean BEV cycles
Group C	5	1.02 ± 0.64	1.42 ± 0.97	40%	1.6
Group D	5 (3 censored)	16.36 ± 2.83	32.27 ± 14.48	100%	15.2
Group E	2 (2 censored)	4.03 ± 5.94	9.650 ± 5.06	100%	7.5

Administration of bevacizumab alone (alone group) was also compared with the combination of bevacizumab and chemotherapy (combination group) for patients with recurrent ovarian cancer, as shown in Table 4. Only the overall response rate showed a non-significant p-value. The remaining variables were significant and revealed that, comparing the bevacizumab alone group and the combination group, the bevacizumab alone group had much shorter progression-free survival (4.19 ± 1.35 vs. 1.02 ± 0.64 months) and overall survival (9.99 ± 2.84 vs. 1.42 ± 0.97 months) and fewer cycles of bevacizumab (4.86 vs. 1.60 cycles) than the combination group.

**Table 4:** Analysis of bevacizumab alone or combined chemotherapy in recurrent treatment in our study. C/T, chemotherapy; PFS, progression-free survival; OS, overall survival; HR, hazard ratio; BEV, bevacizumab; ORR, overall response rate; Ref., reference.

Prior C/T	Median PFS	PFS-HR	Median OS	OS-HR	BEV cycles	ORR
Combine (n = 70)	4.19 ± 1.35	Ref.	9.99 ± 2.84	Ref.	4.86	51.40%
Alone (n = 5)	1.02 ± 0.64	2.56 (1.00–6.56)	1.42 ± 0.97	11.46 (3.95–33.27)	1.6	40%
p-value	0.04	0.050	< 0.001	< 0.001	< 0.001	0.487

In group F, patients with intraperitoneal (IP) administration of bevacizumab, the clinical benefit rate of ascites improvement was 85.6%, as shown in Table 5.

**Table 5:** Outcome of intraperitoneal use of bevacizumab in our study. BEV, bevacizumab.

Group F	Clinical benefit rates	Median BEV cycles	BEV dosage
n = 7	6 (85.6%)	2	5mg/kg

### Safety and adverse events

**Fig 5:**
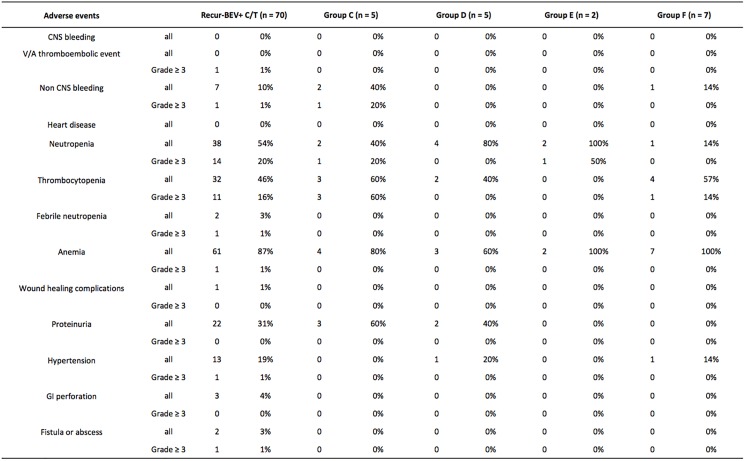
Toxicity of bevacizumab in our study. Adverse events. V/A, venous/artery; CNS, central nervous system; Recur, recurrent ovarian cancer; BEV, bevacizumab; C/T, chemotherapy.

Two deaths related to bevacizumab administration were reported in our study: one was in group B and was due to a suspected venous or arterial thromboembolic event, and the other was in group C and was due to massive gastrointestinal bleeding. Most grade 3 or higher adverse events in the patients of our study were hematologic events. 18% of all women had neutropenia, and 17% had thrombocytopenia. In addition, the percentage of patients with gastrointestinal bleeding was also increased (2%) in our study. Other adverse events included 1% with febrile neutropenia, hypertension, anemia, and bowel fistula. These adverse events mainly occurred in the multiple recurrent ovarian cancer groups (groups A, B, and C). In groups D, E and F, there was only one patient with grade 3 or higher neutropenia and one with grade 3 or higher thrombocytopenia. No other severe adverse events were found in groups D, E, and F. All adverse effects are listed in Fig 5.

## Discussion

From Fig 3, the median progression-free survival of patients with recurrent ovarian cancer treated under Bevacizumab combination with chemotherapy (group A and B) were about 4.17 to 4.24 months upon their initial platinum sensitivity. The median overall survival in both groups were about 9.93 to 10.06 months. One study by Chou in 2005 evaluated Pegylated liposomal doxorubicin for platinum resistant/refractory ovarian cancer, and the median progress-free survival as well as overall survival were 5.4 months and 13.8 months respectively. [22] Another report by Hu in 2015 presented Topotecan in heavily pretreated ovarian cancer, and their median progression-free survival as well as overall survival were 3 months and 20 months respectively.[23] From Fig 4, there were 62 in total 70 patients (88.6%) of group A and B receiving at least two lines of chemotherapy before enrolling into our study. However, the ratio was 69% in study by Chou[22] and 75% by Hu.[23] Thus, much more patients with heavily-pretreated ovarian cancer may lead our outcome lower than others.

No significant differences were found between groups A and B, so the initial platinum sensitivity had no important role in the patients with multiple re-recurrence of ovarian cancer and multiple rounds of chemotherapy. These patients might all be considered to have a platinum-refractory status.

When comparing the response group with the non-response group for bevacizumab combined with chemotherapy to treat recurrent ovarian cancer, the results revealed that ovarian cancer had a worse outcome with bevacizumab than did tubal cancer. In addition, the results of tumor histology showed that the mucinous type had a worse outcome, but the endometrioid type had a better outcome compared to the serous type. Good ECOG performance status (ECOG 0 and 1) had a better outcome with bevacizumab administration, as did lack of ascites, as shown in research by Burger et al. in 2015.[24] In our study, age, prior rounds of chemotherapy, initial CA-125 level, initial FIGO stage, tumor cell grade, and bevacizumab dosage per week had no significant effects on patient outcome or response.

The patients with recurrent ovarian cancer treated with bevacizumab alone were mostly in poor condition and were not suitable candidates for chemotherapy. Therefore, their outcome was poor, and their data were much worse than the data of bevacizumab combined with chemotherapy, as shown in Table 4.

In groups D and E (shown in Table 3), bevacizumab was utilized as front-line adjuvant chemotherapy or neoadjuvant chemotherapy, as in GOG-0218 and ICON7. However, both progression-free survival and overall survival were lower in our study than in those two clinical trials (progression-free survival in groups D, E vs. GOG-0218, and ICON7: 16.36, 4.03 vs. 14.1, 19.0 months, respectively; overall survival: 32.27, 9.65 vs. 39.7, 58 months, respectively).[11–13] Because the data were censored in our study due to a high proportion of living patients (60% in group D; 100% in group E), a better outcome can be expected over time.

There are still unresolved problems related to bevacizumab treatment in ovarian cancer, such as the optimal timing of administration, optimal dosage, best chemotherapy regimen for combination therapy, and the intraperitoneal administration of bevacizumab.[25] Abnormal tumor vasculature showed sprouting vessels with a leaky and tortuous pattern. The increased interstitial fluid pressure compresses intratumoral blood perfusion, and the resulting hypoxic microenvironment makes it difficult to deliver other cytotoxic drugs or radiation therapy.[26] Antiangiogenesis is proven to normalize the tumor vasculature by reducing tumor vascular density as well as permeability, thereby increasing intratumoral perfusion and delivery of chemotherapeutic drugs.[27] However, although increased dosages of antiangiogenic agents have more anti-cancer effects, the excessive vascular regression results in the difficult delivery of cytotoxic drugs. In 2005, Jain RK. demonstrated the vascular normalization time window concerning the optimal timing of chemotherapy combinations according to the balance between the vascular normalization and regression effects of antiangiogenic agents.[28] The biphasic response of increased tumor flow or drug delivery at low doses and decreased flow or delivery at intermediate or high doses, as with bevacizumab in ovarian/esophageal cancer, has been noted with DC101 in breast cancer cells by Huang YH. et al. in 2012[29] and Arjaans M. et al. in 2013,[27] and with vatalanib (PTK787) in lung cancer by Chatterjee S. et al. in 2014.[30] In the present study, the bevacizumab dosage had a significant impact. Low-dose of bevacizumab infusion regimen is not inferior to standard dose of 15mg/m^2^ infusion regimen.

Regarding the timing of bevacizumab administration, in 2007, Dickson PV. et al. reported a higher chemotherapy penetration rate (81%) and tumor growth inhibition (36% of control size) when bevacizumab was administered 1 to 3 days before topotecan compared to concomitant administration or administration 7 days apart.[31] In 2012, Huang reported that treatment with DC101 could reduce the vascular diameter of breast cancer on days 2 and 5, but there was no difference on day 8.[29] In 2015, Ciccolini reported a 5- to 10-day delay to increase tumor perfusion to maximize the effect of chemotherapeutic agents.[32] In the present study, we did not compare the interval between the administration of bevacizumab and chemotherapeutic agents. We compared early and late administration of bevacizumab in combination with chemotherapy in recurrent ovarian cancer, as shown in Fig 4. The early group (only one prior chemotherapy regimen) had a significantly longer progression-free survival than the late group (multiple prior chemotherapy regimens). Although the overall survival, mean number of cycles of bevacizumab administration, and overall response rate showed no significant differences, the earlier administration of bevacizumab in combination with chemotherapy still plays a role in influencing a better outcome in recurrent ovarian cancer. However, based on the experience reported by Lai A et al. in 2013 of using bevacizumab in recurrent glioblastoma, delayed use of bevacizumab is not associated with diminished efficacy and therefore is preferred for those patients with glioblastoma because there is a fixed survival after bevacizumab initiation.[33]

In addition, in 2012, Chauhan VP. et al. reported that vascular normalization by antiangiogenic agents can improve vascular permeability and subsequent drug delivery of 10- to 12-nm particles but not of larger nanoparticles.[34] In 2013, Stylianopoulos and Jain developed a model to combine vascular normalization and stress alleviation to reveal an optimal perfusion region with acceptable permeability for drug delivery. They reported that the region is much more suitable for drugs 10 nm in diameter with low affinity, such as nab-paclitaxel, than for 60 nm or 120 nm particles, such as PEGylated liposomal doxorubicin.[35] Thus, the size of the particles might determinate the suitable chemotherapy regimen for combination therapy. In 2015, Chan et al. suggested a longer overall survival with bevacizumab combined with PEGylated liposomal doxorubicin (20.4 months) or taxanes (20.2 months) than with gemcitabine (14.1 months), topotecan (13 months), or cyclophosphamide (13 months).[36] However, there were no significant differences in overall survival between different chemotherapy regimens combined with bevacizumab in our study.

There are still other possible sources of drug resistance related to antiangiogenic agents, such as tumor hypoxia and vessel co-option. Increased tumor hypoxia caused by neovascularization blockade by antiangiogenic agents leads to elevation of hypoxia-inducible factors (HIFs). HIF 1a and/or 2a can promote cancer cell survival and maintain the cancer stem cell microenvironment.[37] In 2012, Conley SJ. et al. found increased intratumoral hypoxia as well as cancer stem cells in human breast cancer xenografts in patients treated with sunitinib and bevacizumab.[38] In 2014, Pham et al. reported an improved response rate with bevacizumab combined with CRLX101, a nanoparticle-containing camptothecin that reduces HIF upregulation.[39] In 2016, Kuczynski EA. et al. revealed vessel co-option of hepatocellular carcinoma to hijack the vessels from other organs during treatment with sorafenib.[40]

IP bevacizumab for clinical ascites might also have some effects, as revealed in Table 5, and the associated adverse events are few. As Jiang et al. reported in 2016, intrapleural and intraperitoneal administration of bevacizumab plus cisplatin for patients with malignant pleural effusion and ascites can lead to a better quality of life and objective response rate than treatment with cisplatin alone.[41]

## Conclusion

In conclusion, considering adverse events, safety, and patient outcomes, earlier administration of bevacizumab in combination with chemotherapy resulted in advantages in patients with recurrent ovarian cancer. In addition, the intraperitoneal administration of bevacizumab obviously improved the patients’ clinical ascites. Of course, the sample size of our study was small and uni-centered. Besides, our study still had some common limitation of retrospective studies such as lack of randomization to represent the general population, selection bias while data collection, and difficult to have better evidence than prospective study when evaluation of the results. Further investigation of bevacizumab in recurrent ovarian cancer is needed to determine which chemotherapy regimen is optimal for patient outcome and response.

## Supporting information

S1 Table All patient list with all parameters, survivals, and toxicity. (XLSX)
